# Retraction: Neoadjuvant Therapy for Esophageal Adenocarcinoma in the Community Setting—Practice and Outcomes

**DOI:** 10.3389/fonc.2019.00324

**Published:** 2019-04-24

**Authors:** Frontiers Editorial Office

The journal retracts the 18 July 2017 article cited above.

During a recent review by the Creighton University Institutional Review Board that approved this study, it was found that the authors utilized and included some datasets that were not IRB-approved. Therefore, the study did not receive full ethics committee approval and is being withdrawn.

The authors concur with the retraction and sincerely regret any inconvenience this may have caused to the reviewers, editors, and readers of Frontiers in Oncology.

